# The Consumption and Diversity Variation Responses of Agricultural Pests and Their Dietary Niche Differentiation in Insectivorous Bats

**DOI:** 10.3390/ani14050815

**Published:** 2024-03-06

**Authors:** Dan Zhu, Yingying Liu, Lixin Gong, Man Si, Qiuya Wang, Jiang Feng, Tinglei Jiang

**Affiliations:** 1 Jilin Provincial Key Laboratory of Animal Resource Conservation and Utilization, Northeast Normal University, 2555 Jingyue Street, Changchun 130117, China; zhud969@nenu.edu.cn (D.Z.); liuyy777@nenu.edu.cn (Y.L.); gonglx216@nenu.edu.cn (L.G.); sim663@nenu.edu.cn (M.S.); wangqiuya123@outlook.com (Q.W.); 2 Key Laboratory of Vegetation Ecology of Education Ministry, Institute of Grassland Science, Northeast Normal University, 2555 Jingyue Street, Changchun 130117, China; 3 College of Life Science, Jilin Agricultural University, 2888 Xincheng Street, Changchun 130118, China

**Keywords:** bats, DNA metabarcoding, agricultural pests, diet, pest suppression, functional response, niche partitioning, species traits

## Abstract

**Simple Summary:**

In this study, we performed DNA metabarcoding to examine prey composition and pest diversity in the diets of four insectivorous species of bats. Then, we evaluated the correlation between bat activity and insect resources and assessed dietary niche similarity and niche breadth among species and factors that influence prey consumption in bats. Additionally, we explored the functional response between these predators and their prey, understanding how bat feeding behavior adapts to the availability of different pest species. We proved that bats provide vital pest consumption services in agricultural ecosystems and their diet included arthropods from 23 orders and 200 families. Moreover, bats responded to the availability of insects. For example, a higher abundance of insects, especially Lepidoptera, and a higher insect diversity led to an increase in the duration of bat activity. In areas with more abundant insects, the number of bat passes also increased. Our results suggested that dietary niche differentiation promotes the coexistence of different bat species and increases the ability of bats to consume insect prey and agricultural pests. Our findings provide greater insights into the role of bats that prey on agricultural pests and highlight the importance of combining bat conservation with integrated pest management.

**Abstract:**

Insectivorous bats are generalist predators and can flexibly respond to fluctuations in the distribution and abundance of insect prey. To better understand the effects of bats on arthropod pests, the types of pests eaten by bats and the response of bats to insect prey need to be determined. In this study, we performed DNA metabarcoding to examine prey composition and pest diversity in the diets of four insectivorous species of bats (*Hipposideros armiger*, *Taphozous melanopogon*, *Aselliscus stoliczkanus*, and *Miniopterus fuliginosus*). We evaluated the correlation between bat activity and insect resources and assessed dietary niche similarity and niche breadth among species and factors that influence prey consumption in bats. We found that the diets of these bats included arthropods from 23 orders and 200 families, dominated by Lepidoptera, Coleoptera, and Diptera. The proportion of agricultural pests in the diet of each of the four species of bats exceeded 40% and comprised 713 agricultural pests, including those that caused severe economic losses. Bats responded to the availability of insects. For example, a higher abundance of insects, especially Lepidoptera, and a higher insect diversity led to an increase in the duration of bat activity. In areas with more abundant insects, the number of bat passes also increased. The dietary composition, diversity, and niches differed among species and were particularly significant between *H. armiger* and *T. melanopogon*; the dietary niche width was the greatest in *A. stoliczkanus* and the narrowest in *H. armiger*. The diet of bats was correlated with their morphological and echolocation traits. Larger bats preyed more on insects in the order Coleoptera, whereas the proportion of bats consuming insects in the order Lepidoptera increased as the body size decreased. Bats that emitted echolocation calls with a high peak frequency and duration preyed more on insects in the order Mantodea. Our results suggest that dietary niche differentiation promotes the coexistence of different bat species and increases the ability of bats to consume insect prey and agricultural pests. Our findings provide greater insights into the role of bats that prey on agricultural pests and highlight the importance of combining bat conservation with integrated pest management.

## 1. Introduction

Herbivorous insect pests are a serious threat to agriculture as they greatly affect plant reproduction and reduce the biomass and distribution of crops [1]. Insect pests cause 25–50% of crop loss in the world [2]. They have wide-ranging effects on the agricultural production of food, fiber, timber, and livestock [3,4]. Traditional crop protection predominantly relies on chemical control to prevent or mitigate the damage caused by pests. The cost of direct pesticide control exceeds USD 10 billion per year [5]. Over time, the overuse of chemical pesticides has decreased crop quality [6], increased pesticide residue and pathogen resistance, worsened environmental pollution, and significantly affected human health [7]. Pest control services provided by natural enemies have become popular and may be an effective and less environmentally damaging means of suppressing pests than chemical pesticides [8]. Therefore, integrated pest management needs to be vigorously promoted [9].

Several natural predators of pests (generalists and specialists) are present in agricultural ecosystems [10]. Generalist predators are usually opportunistic predators; persistence in foraging time allows generalist predators to prey on different pests following an outbreak, track sudden pest invasions, and respond to changes in available pest resources [11,12,13,14,15]. Therefore, generalist predators serve as biological factors that play a crucial role in regulating the dynamic processes of harmful arthropods [14,16]. In recent years, the demand for the use of pest control services provided by natural predators of insects has increased, necessitating an assessment of the dietary composition of various predators and their interactions with pests. Such information can facilitate a better understanding of the trophic relationships between predator and prey in agricultural ecosystems, which can help propose better pest management solutions.

Bats are the second-largest group of mammals, with more than 1460 species. Most of them are generalist predators of pests in agricultural ecosystems [17]. Most bat species are insectivores and consume a large number of insects (up to 70–84% of their body mass) every night [18], and sometimes up to 100% [19]. Bats provide valuable ecosystem services by significantly affecting crop pest populations and are now considered to be economically important [20,21]. For example, *Tadarida brasiliensis* preys on pests of cotton and can reduce the need to use pesticides. The estimated value was about USD 741,000 for cotton fields in south-central Texas, where the bat *T. brasiliensis* is responsible for saving up to 15% of the final value each year as a natural controller of the cotton bollworm (*Helicoverpa armigera*) [22]. Annual costs of around USD 613/ha in the macadamia orchards of South Africa are avoided because of the presence of bats [23]. Insectivorous bats also have a wide range of foraging areas and can feed on newly available resources, showing a functional response to pest outbreaks [12,13]. Functional response is the response of the predation rate or activity of each predator to variation in prey density, i.e., the response of predators to prey [24]. For example, in a study, bat activity and species richness increased as cotton growth progressed, reaching a peak when pest abundance was high. Bat activity and foraging were higher on nights in which insect abundance was high [25]. Therefore, studying the functional response of insectivorous bats to insects can help understand the dietary relationships and foraging patterns of bats in the ecosystem, leading to a better understanding of the pest control services provided by bats. Studies on pest suppression services provided by bats are limited to the Neotropics and Europe; thus, studies from Africa and Asia are lacking [26]. Some studies on the diet of insectivorous bats have been conducted in China [27,28,29]; however, studies on the interaction between bats and pests in combination with the diet of bats are rare and further investigation is needed.

Insectivorous bats usually co-occur in the same cavities or areas with ecologically similar characteristics. The ecological niches of sympatric species may overlap, resulting in some degree of competition for limited resources [30]. Therefore, different species of bats adopt different foraging strategies or dietary preferences to co-exist [31]. The morphological and echolocation traits of bats may influence their consumption of insects [3,32]. To maximize the capture of suitable insect prey, insectivorous bats can adjust their foraging behavior [33]. A smaller degree of overlap in resource use between species (niche differentiation) can increase the diversity and abundance of insects (including pests) consumed by bats, which in turn can significantly affect agroecosystems [34,35]. However, traditional methods of diet analysis generally underestimate the diversity of prey consumed [36], which makes it difficult to differentiate between dietary niches among species [37]. Using DNA metabarcoding, a large number of samples can be processed quickly, and the diets of animals can be evaluated at the species level [38,39]. Thus, DNA metabarcoding data can help to elucidate the mechanism of dietary niche differentiation in different species of bats in the same area.

In this study, we assessed the predation on insect pests by insectivorous bats in crop plantations, investigated the composition of background insect resources, evaluated the activity of bats in the study area, and performed DNA metabarcoding to determine prey composition and pest diversity in the diets of four common species of insectivorous bats *(H. armiger*, *T. melanopogon*, *A. stoliczkanus*, and *M. fuliginosus*). We also determined the correlation between bat activity and insect resources and assessed dietary niche similarity and breadth among species and factors that influence prey consumption in bats. We hypothesized that bats prey on different species of agricultural pests and that their predation activity and dietary differences are related to changes in insect resources and the traits of bat species. We predicted the following: (a) the prey composition in the diet of bats includes different types of agricultural pests; (b) bat activity is related to changes in insect resources; (c) dietary niches are different among the four bat species; and (d) the morphological and echolocation traits of bats can affect their prey choice and consumption. We aimed to provide novel insights into predation on pests by insectivorous bats, identify factors that influence the consumption of prey pests by bats, and show the effectiveness of bats in integrated pest management.

## 2. Materials and Methods

### 2.1. Study Site and Insect Survey

This study was conducted in Pu’er City, Yunnan Province (22°29′–22°49′ N and 100°38′–100°56′ E), where the land is mainly used for agriculture. Maize and rice are the main crops in the region. The crop is harmed by pests throughout the year. The region has a subtropical plateau monsoon climate with average annual temperatures around 18.2 °C and a mean annual rainfall of 1700 mm per year. We surveyed the activity and diet of bats and local insect resources from summer to early autumn during the crop-growing season in 2022.

**Figure 1:**
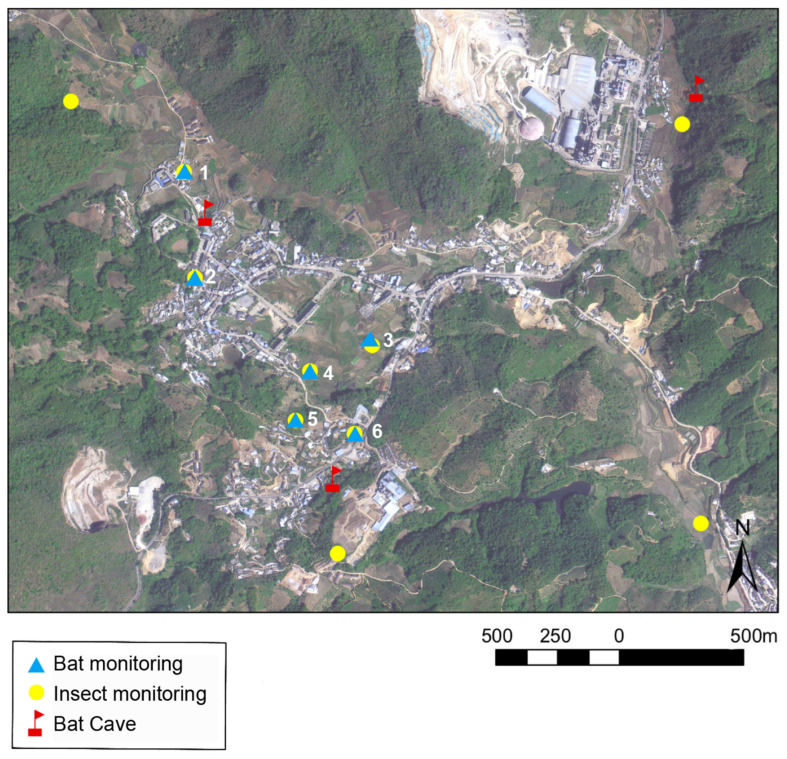
The locations of bat caves, bat monitoring sites, and insect trap sites in the study area are shown on the map.

The insect survey was conducted from June to mid-September 2022. We alternately placed light insect traps (6 W portable heath moth trap) to lure insects at 10 randomly selected internal sample sites in the crop field (Figure 1). The insect trap was placed 1 m above the ground every night from 20:00 PM to 3:00 AM the following morning. The collected insects were initially identified based on their morphological traits, and the number of insects was counted at the order level. All samples were preserved individually in 95% ethanol. We collected data for 40 nights from 10 sites during the sampling time. To ensure the accuracy of insect identification, DNA metabarcoding was used. We mixed the collected insects according to the sample sites, and 33 samples were used for molecular analysis, with four samples each from sample sites 4, 5, and 10, and three samples each from the remaining sample sites.

### 2.2. Passive Acoustic Monitoring of Bats

We placed six AudioMoth acoustic detectors [40] more than 3 m from the ground to record the activity of bats from sunset to sunrise the following day [41]. All monitoring sites were close to the insect traps (Figure 1). We set the detectors to record sequences 55 s long at a sampling rate of 250 kHz. We monitored data for 31 nights and obtained acoustic effective wave data for 25 days. Then, we used Avisoft SASLAB Pro (version 5.3.01, Avisoft Bioacoustics, Berlin, Germany) to process the collected acoustic files. We discarded insect noise and non-biological sounds. To characterize the activity of bats, the total number of bat passes and the duration of bat activity in the files at each sample site were manually counted. Bat pass was defined as a single or several bat calls emitted during a fixed interval (5 s in this study). The duration of bat activity was defined as the activity of each bat (in seconds) monitored within the detection range of the acoustic detector [42,43].

### 2.3. Field Investigations and Dietary Sampling of Bats

We identified three roost caves with a large population and different species of bats. The distances between the three caves and the light trap were different. The average linear distance between the farthest roost and the light trap was 1.84 km, and the average linear distance between the nearest roost and the light trap was only 0.61 km. Bats were captured using mist nets spread at cave entrances when the bats returned from foraging (between 9:00 PM and 6:00 AM). Samples were collected at 15-day intervals in three different caves to minimize interfering with the bats. From June to mid-September 2022, we collected samples 11 times and obtained 203 bat individuals from 14 species. The bats were captured using mist nets as they entered the caves after foraging. We identified the species of bats after trapping them. Each individual was placed in a clean and sterilized waterproof paper bag and held for 1–2 h to collect fecal samples. Each fecal sample was placed in a 2 mL cryopreservation tube and stored at −80 °C until DNA extraction. After collecting the fecal samples, we measured the morphological parameters of each individual for further analyses, including the length of the right forearm and the body mass (to the nearest 0.01 mm and gram, respectively). The forearm length was measured using an electronic Vernier caliper (111–101V-10G, Guanglu Ltd., Shenzhen, China), and an electronic balance (BSA4202S, Sartorius Ltd., Shandong, China) was used to measure the body mass. The forearm length of each individual was measured thrice, and the mean value was used in the analyses [44]. Using the data on forearm length and body mass, the forearm mass index (FMI) was calculated [45]. All bats were released after sample collection. We selected fecal samples of the four insectivorous bat species with the greatest abundance in the region for dietary analysis. In total, 66 samples were used for molecular analysis, including 16 samples of *H. armiger*, 17 samples of *T. melanopogon*, 17 samples of *A. stoliczkanus*, and 16 samples of *M. fuliginosus*. The acoustic parameters of echolocation calls, including peak frequency, bandwidth, and duration, of the four bat species were obtained from previous studies [46,47,48].

### 2.4. DNA Extraction, PCR Amplification, and Sequencing

The DNA was extracted from fecal samples using a QIAamp DNA Stool Mini Kit (Qiagen, Manchester, UK), following the manufacturer’s protocol. The quality and concentration of DNA were determined by 1.0% agarose gel electrophoresis using a NanoDrop^®^ ND-2000 spectrophotometer (Thermo Scientific Inc., Waltham, MA, USA). The hypervariable regions of the cytochrome oxidase I (COI) marker located within the conventional barcode region were amplified using the primer pairs LCO-1490 (5’-GGTCAACAAATCATAAAGATATTGG-3′) and ZBJ-ArtR2cR (5’-WACTAATCAATTWCCAAATCCTCC-3′) [49,50] and an ABI GeneAmp^®^ 9700 PCR thermocycler (ABI, Los Angeles, CA, USA). The PCR reaction mixture included 10 µL of 2 × Pro Taq, 0.8 µL of forward primer (5 µM), 0.8 µL of reverse primer (5 µM), 10 ng of template DNA, and ddH_2_O to a final volume of 20 µL. The PCR amplification cycling conditions were as follows: initial denaturation at 95 °C for 10 min, followed by 30 cycles of denaturation at 95 °C for 30 s, annealing at 55 °C for 30 s, extension at 72 °C for 60 s, single extension at 72 °C for 10 min, and storage at 10 °C. All samples were amplified in triplicate. The PCR product was extracted from 2% agarose gel and purified using the AxyPrep DNA Gel Extraction Kit (Axygen Biosciences, Union City, CA, USA) and quantified using QuantiFluor-ST (Promega, Madison, WI, USA), following the manufacturer’s protocol. The products were sequenced on an Illumina MiSeq PE300 platform (Illumina, San Diego, CA, USA) following the standard protocols provided by Majorbio Bio-Pharm Technology Co., Ltd. (Shanghai, China). The insect samples were treated in the same way as the fecal samples.

### 2.5. Sequence Analysis and Taxonomic Identification

Raw FASTQ files were de-multiplexed using an in-house Perl script, quality-filtered using fastp version 0.19.6 [51], and merged using FLASH version 1.2.7 with the following criteria [52]: (i) The 300 bp reads were truncated at any site receiving an average quality score of <20 over a 50 bp sliding window. Truncated reads shorter than 50 bp and reads containing ambiguous characters were discarded. (ii) Only overlapping sequences longer than 10 bp were assembled according to their overlapped sequence. The maximum mismatch ratio of the overlap region was 0.2. Reads that could not be assembled were discarded. (iii) The samples were distinguished according to the barcode and primers, and the sequence direction was adjusted, with exact barcode matching and two nucleotide mismatches in primer matching. Then, the optimized sequences were clustered into operational taxonomic units (OTUs) with a 97% sequence similarity level using UPARSE 7.1 [53,54]. The most abundant sequence for each OTU was selected as the representative sequence. To ensure the accuracy and reliability of DNA sequences, the sequences that appeared in less than two PCR replicates were removed. Sequences that were identical to those detected in the extraction and library blanks of the corresponding processing batch of each sample were removed. Taxonomic identification was performed by aligning the representative sequence from each OTU to reference sequences in the GENBANK NT and Barcode of Life Data Systems (BOLD) databases (www.boldsystems.org, accessed on 1 July 2023). Classification at the order and family levels was assigned at >95% and >96.5% identity values, respectively. When the identity values between the query and reference sequences were > 98%, species-level taxonomy was assigned [55]. Insect samples were handled similarly to fecal samples.

### 2.6. Sympatric Bat Dietary Analysis and Niche Differentiation Exploration

We calculated the relative read abundance (RRA) and frequency of occurrence (FOO) of prey order in the diets of four bat species (*H. armiger*, *T. melanopogon*, *A. stoliczkanus*, and *M. fuliginosus*) and background insect resources [21]. We visualized the dietary composition of bats and local insect resources using the R package metacode (version 0.3.6) [56]. The R package ‘VennDiagram (version 1.7.3)’ [57] was used to display the common and unique prey OTUs of the diets of the four species of bats. Additionally, the economic effects of the identified insect species were obtained from various sources and published studies on insect taxonomy [58,59,60] and divided into three groups based on whether the insects were harmful to crops; these groups included non-pests, agricultural pests, and other pests. Other pests include forestry pests and pests that can transmit diseases among humans and livestock. The species richness and Shannon diversity indices for the identified prey of each bat individual among the four common species were evaluated using the R package ‘vegan (version 2.6-4)’ [61]. Nonmetric multidimensional scaling (NMDS) with the Bray–Curtis distance was used to determine the degree of similarity in diet among the four species using the R package ‘vegan (version 2.6-4)’ [61]. We assessed the degree of dietary overlap among the four bat species using Pianka’s (1974) niche overlap indices. The index ranges from 0 (indicating no overlap) to 1 (indicating complete overlap), with values >0.6 or <0.4 generally considered to be ecologically significant correlations representing high or low levels of dietary overlap, respectively [62]. Dietary niche width at the population level for each bat species was computed by applying Levins’ index [63].

### 2.7. Statistical Analyses

To assess the determinants of bat activity, 10 factors were selected as independent variables, including the Shannon diversity index of insects (HI), the Simpson diversity index of insects (DI), the richness of insects (RI), the abundance of insects (AI), the richness of Lepidoptera (LepR), the abundance of Lepidoptera (LepA), the richness of Coleoptera (ColR), the abundance of Coleoptera (ColA), the richness of Diptera (DipR), and the abundance of Diptera (DipA), to construct two linear models (LMs); the duration of bat activity and the number of bat passes were used as dependent variables, respectively. We excluded the multicollinearity between variables using the package ‘fmsb (version 0.7.6)’ [64]. For the duration of bat activity, factors with multicollinearity included AI with DipA, ColA with LepA, DI with HI, and RI with ColA. Thus, the three factors, DipA, ColA, and DI, were excluded from the linear model. For the number of bat passes, factors with multicollinearity included DI with HI, and AI with LepA, ColA, LepR, and DipA. Thus, the four factors, LepA, ColA, LepR, and DipA, were excluded from the linear model. The package MuMIn (version 1.47.5) [65] and the AIC method were used for selecting the optimal model. The LM with the smallest AIC value was identified as the best-fitting model. The LMs were validated using the R package ‘DHARMa (version 0.4.6)’ [66]. Residuals were created using the simulateResiduals function with 1000 simulations. Residual dispersion was assessed using the testDispersion function, and the residuals were found to follow a normal distribution using the testUniformity function.

We compared differences in dietary niche width among the four species of bats by conducting a Kruskal–Wallis *H* test. An ANOVA was performed to compare the species richness and Shannon diversity indices of the diet of the bats. The dietary differences among bats were tested using a permutational multivariate analysis of variance (PERMANOVA) using the function adonis based on 999 permutations in the R package ‘vegan (version 2.6-4)’ [61]. We tested for pairwise differences between species using the pairwise Adonis package (version 0.4-1) and performed Bonferroni correction for multiple comparisons [67]. We also conducted an analysis of similarities (ANOSIM) to test whether the Jaccard distance of prey composition was greater between bat species than within bat species [68].

To assess how morphological and echolocation traits of bats affect their prey choice and consumption, we selected three morphological variables (body mass, forearm length, and FMI) and three acoustic variables (peak frequency, bandwidth, and duration) to evaluate their relative contributions to dietary niche differences by OTUs at the order and family levels using the R package ‘stats (version 4.3-1)’ [69]. Then, the package ‘pheatmap (version 1.0.12)’ [70] in R was used to visualize the results. All statistical analyses were conducted using R 4.3.1 [71].

## 3. Results

### 3.1. Insect Abundance and Bat Activity

**Figure 2:**
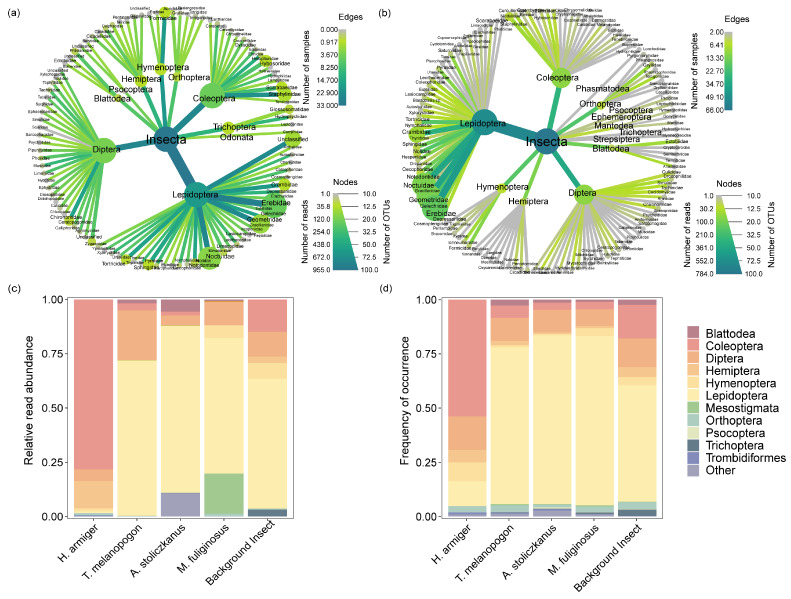
The proportion of insects in the diet of the four bat species and the proportion of local background insects. (**a**) Regional arthropod resources. (**b**) Arthropods consumed by the four common species of bats. (**c**) Relative read abundance (RRA) and (**d**) frequency of occurrence (FOO) of prey orders in the diet of the four bat species and local background insects.

We collected 6802 insect samples from 10 light traps. Additionally, 1203 OTUs were generated via sequence processing and the taxonomic identification of local background insects; 966 OTUs corresponded to 597 species of arthropods from 13 orders and 124 families (Figure 2a). Lepidoptera (53.79%) dominated the available insect resources, followed by Coleoptera (15.57%) and Diptera (13.16%) (Appendix A).

**Figure 3:**
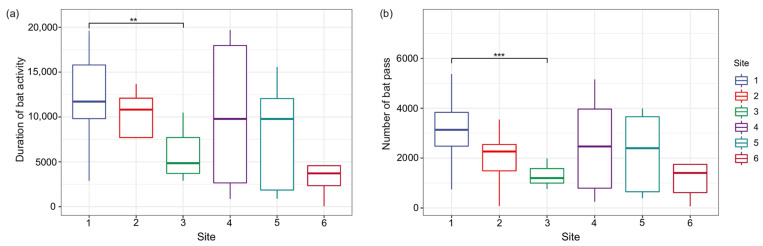
The duration of bat activity (**a**) and the number of bat passes (**b**) in six sample sites. ** *p* < 0.01 and *** *p* < 0.001.

We recorded 115,686 passes at six sample sites over 25 nights, with an activity time of 446,214 s. The number of bats that passed through the most active sites was 3105 per night on average, and the activity time was 12,282.2 s. The least active sites were visited only 1300 times per night on average, and the activity time was 5867.914 s. The differences in the duration of bat activity and the number of bat passes were significant only between sites 1 and 3 (Figure 3).

### 3.2. Prey Composition and Pest Diversity in the Diet of Bats

**Figure A1:**
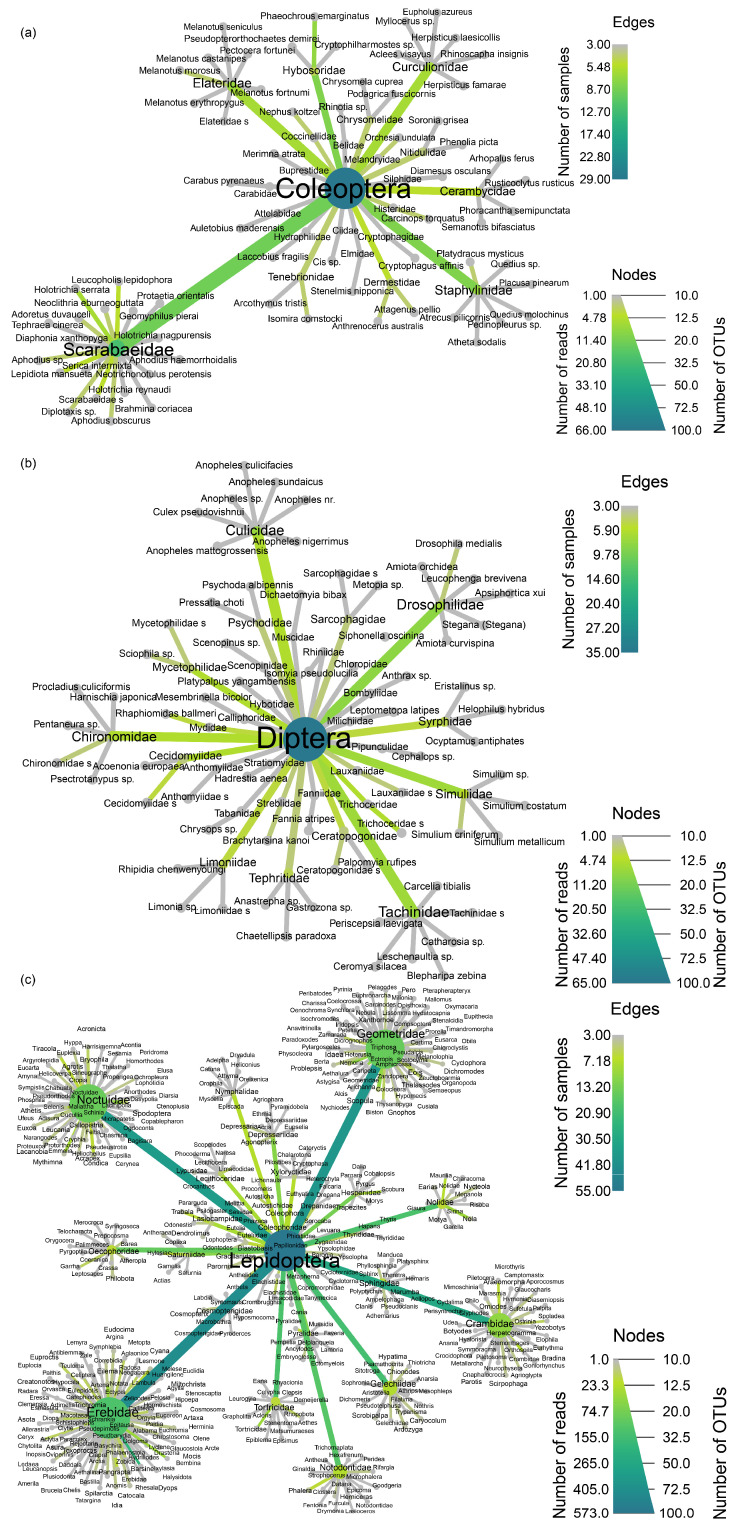
Dietary composition of the four bat species. (**a**) Coleopteran prey in the diet. (**b**) Dipteran prey in the diet. (**c**) Lepidopteran prey in the diet.

We analyzed fecal samples of 66 bats belonging to four species: *H. armiger* (*n* = 16), *T. melanopogon* (*n* = 17), *A. stoliczkanus* (*n* = 17), and *M. fuliginosus* (*n* = 16). In total, 2010 OTUs were obtained after sequence processing and taxonomic identification. Among them, 1732 corresponded to 1412 arthropod species from 23 orders and 200 families (Figure 2b). In general, the diet of bats was dominated by Lepidoptera (71.51%), Diptera (10.06%), and Coleoptera (7.49%). Within the Lepidopterans, Erebidae, Noctuidae, and Geometridae were the most represented families. Scarabaeidae and Elateridae were the dominant families of Coleoptera, whereas Diptera was mostly represented by the families Culicidae and Tachinidae. The remaining prey detected mostly contained species of Hymenoptera, Orthoptera, Hemiptera, and Blattodea. In contrast to the other three bat species, *H. armiger* primarily preyed on Coleoptera (53.85%) rather than Lepidoptera (11.54%). We performed a basic categorization of the prey consumed by the four species of bats and found that a large proportion of the prey consisted of pests (Table 1; Appendix A; Appendix B, Figure A1). Among the prey of *H. armiger*, the percentage of pests was quite high (72.12%), of which agricultural pests comprised 50.00%. We also found that 77.17% of the prey of *T. melanopogon* consisted of pests, among which 46.65% were agricultural pests. The diet of *A. stoliczkanus* had a total pest percentage of 75.44%, and 42.17% of those were agricultural pests. The percentage of pests found in the diet of *M. fuliginosus* was 71.87%, with agricultural pests accounting for 45.47%. Some non-pest arthropod species also appeared frequently in the diet of the bats (Table 1; Appendix A).

### 3.3. Factors Influencing Bat Activity

**Figure A2:**
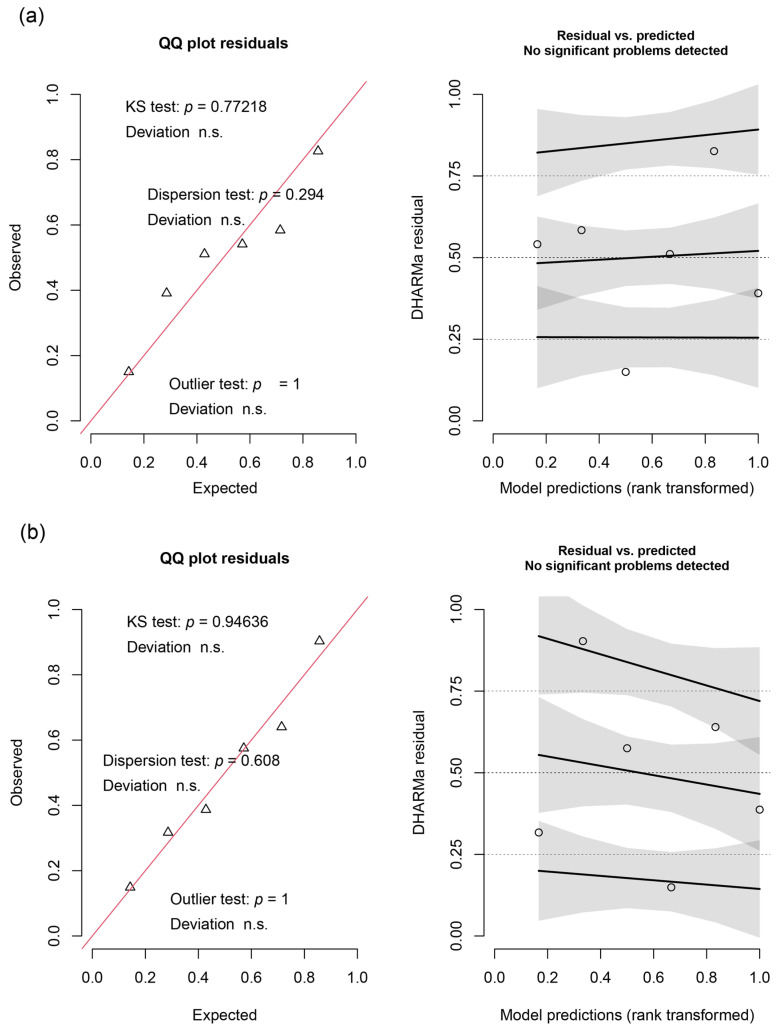
The distribution pattern of the residuals of the two LMs. (**a**) The duration of bat activity. (**b**) The number of bat passes. The left panel is a QQ plot used to detect overall deviations from the expected distribution, with tests for correct distribution (KS test), dispersion, and outliers. The right panel shows the residuals against the predicted values and a quantile regression with 0.25, 0.50, and 0.75 quantile lines across the plot.

The best-fitting model (smallest AIC) of the number of bat passes included two predictors, viz., AI and DipR, and the number of bat passes was significantly positively correlated with AI (Table 2). The best-fitting model of the duration of bat activity included three predictors, viz., HI, LepA, and AI, and the duration of bat activity was significantly affected by HI, LepA, and AI (Table 3). The residuals of the two LMs were normally distributed (Appendix B, Figure A2). Thus, the abundance and diversity of insects, as well as the abundance of Lepidoptera, were high, which led to a corresponding increase in bat activity (the number of bat passes and the duration of bat activity).

### 3.4. Dietary Diversity, Overlap, and Niche Width of Bats

**Figure 4:**
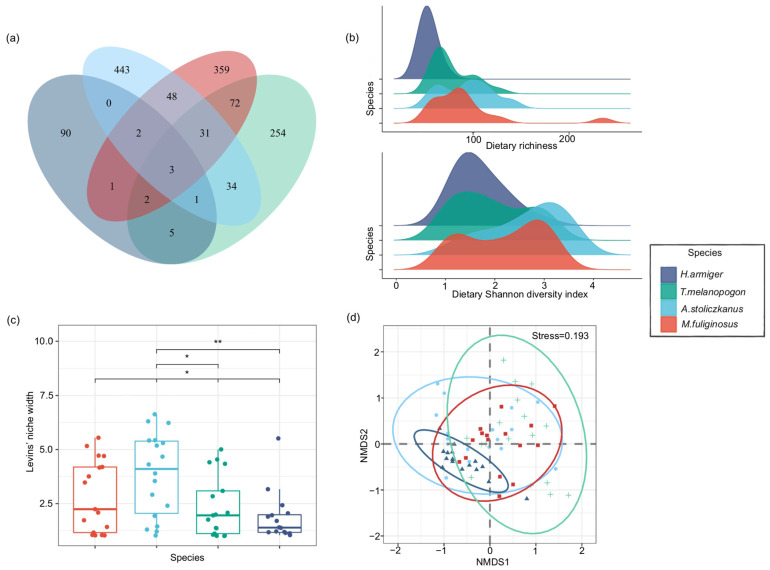
Dietary diversity, overlap, and niche width of the four species of bats. (**a**) The Venn diagram shows the overlapping numbers of consumed OTUs among species. (**b**) Dietary richness and Shannon diversity. (**c**) Dietary niche width. (**d**) The NMDS diagram shows the degree of overlap (similarity) of the diet of four bat species; ellipses represent 95% confidence intervals. * *p* < 0.05 and ** *p* < 0.01.

We found that five prey OTUs overlapped between *H. armiger* and *T. melanopogon*, and one prey OTU overlapped between *H. armiger* and *A. stoliczkanus*. However, there were no overlapping prey OTUs between *H. armiger* and *M. fuliginosus*. *T. melanopogon* and *A. stoliczkanus* had 34 overlapping prey OTUs, whereas *T. melanopogon* and *M. fuliginosus* had 72 overlapping prey OTUs. Between *A. stoliczkanus* and *M. fuliginosus*, 48 prey OTUs overlapped (Figure 4a). The dietary richness and Shannon diversity index varied significantly among the four bat species (*p* < 0.01). *A. stoliczkanus* had the highest dietary richness (62.9 ± 34.2) and Shannon diversity index (1.6 ± 0.8). The dietary richness and Shannon diversity index of *M. fuliginosus* were 60.3 ± 54.3 and 1.3 ± 0.8, while those of *T. melanopogon* were 43.8 ± 26.8 and 0.9 ± 0.7, respectively. *H. armiger* had the lowest dietary richness (11.9 ± 8.3) and Shannon diversity index (0.7 ± 0.5) (Figure 4b). The dietary niche width among species was significant (*p* < 0.05). The results of the pairwise comparison showed that significant differences occurred in dietary niche width between *M. fuliginosus* and *H. armiger*, between *A. stoliczkanus* and *T. melanopogon*, and between *A. stoliczkanus* and *H. armiger* (Figure 4c). The NMDS analysis showed that the samples were largely partitioned by bat species, suggesting that dietary composition varied among bat species (stress = 0.193 < 0.2; Figure 4d). The PERMANOVA results indicated that significant differences occurred in the dietary composition of *H. armiger* and *T. melanopogon* (Pseudo-F = 3.421, *R^2^* = 0.099, *p* = 0.006; Appendix B, Table A1), which implies that the significant factors that accounted for variances in dietary niches among the four bats were *H. armiger* and *T. melanopogon*. Pianka’s niche-overlap index was 0.158 between *H. armiger* and *T. melanopogon*, 0.627 between *H. armiger* and *A. stoliczkanus*, and 0.667 between *H. armiger* and *M. fuliginosus*. Pianka’s niche-overlap index for the overlap of *T. melanopogon* with *A. stoliczkanus* and *M. fuliginosus* was 0.302 and 0.351, respectively; the index for the overlap between *A. stoliczkanus* and *M. fuliginosus* was 0.677 (Table 4). The indices for niche overlap between *T. melanopogon* and the other three species of bats were below 0.4, indicating a low degree of dietary overlap with the other three species of bats. In contrast, the indices for the niche overlap among *H. armiger*, *A. stoliczkanus*, and *M. fuliginosus* were above 0.6, suggesting a higher degree of dietary overlap among them. The results of the ANOSIM also showed that the distance in dietary communities among samples was greater between species than within species (*R* = 0.1976, *p* = 0.001, permutation = 999).

### 3.5. Factors Influencing Prey Consumption by Bats

**Figure 5:**
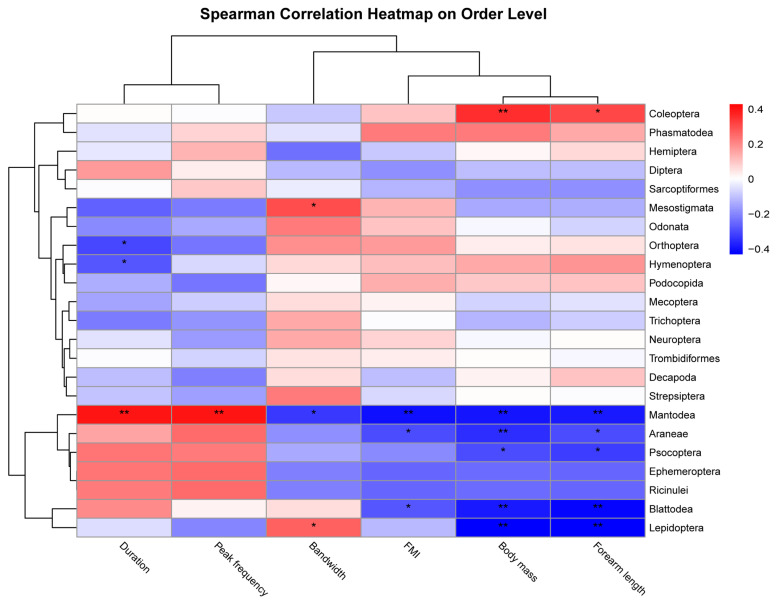
Spearman’s correlation analyses were performed to determine the relationship of the OTUs of bat prey at the order level with different morphological and acoustic parameters of bats; * *p* < 0.05 and ** *p* < 0.01.

**Figure A3:**
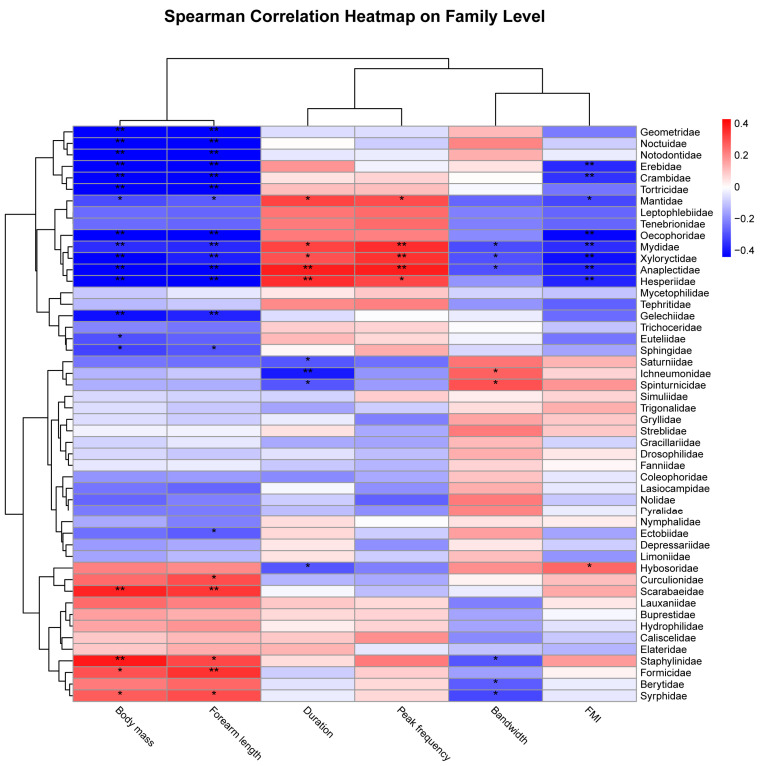
Spearman’s correlation was performed to determine the relationship of the OTUs of the bat prey at the family level with different morphological and acoustic parameters of the bats; * *p* < 0.05 and ** *p* < 0.01.

The results of Spearman’s correlation analysis indicated that larger bats consumed more Coleoptera than smaller bats (Figure 5). Three families in Coleoptera (Scarabaeidae, Staphylinidae, and Curculionidae) showed a significant positive correlation with bat body mass and forearm length (Appendix B, Figure A3). Smaller bats consumed more Mantodea, Araneae, Psocoptera, Lepidoptera, and Blattodea. Noctuidae, Geometridae, and Crambidea, which contained a large number of pest species, were significantly negatively correlated with the size of bats (*p* < 0.01; Appendix B, Figure A3). We also found that the echolocation traits (duration, peak frequency, and bandwidth) of bats were also related to their prey. Bats with a higher peak frequency, longer duration, and narrow bandwidth hunted more Mantodea. The duration of echolocation calls, however, showed a significant negative correlation with Orthoptera and Hymenoptera. The bandwidth also had a significantly positive correlation with Lepidoptera and Mesostigmata (*p* < 0.05; Figure 5).

## 4. Discussion

In this study, we found that the diet of the four species of bats comprised a high proportion of pests, with agricultural pests occupying a relatively large proportion. Our results showed that the activity of bats was associated with changes in insect resources. The diets of the four species of bats were different; the difference was significant between *H. armiger* and *T. melanopogon*, and the dietary niche width was the greatest in *A. stoliczkanus* and the narrowest in *H. armiger*. Across all individuals, larger bats consumed more Coleoptera than smaller bats; the peak frequency, duration, and bandwidth of echolocation calls affected predation on Mantodea, Lepidoptera, Orthoptera, and Hymenoptera. These results are consistent with our hypothesis and predictions. Our findings suggest that insectivorous bats prey on numerous pests in crop plantations, and multiple factors influence the consumption of prey insects by bats. We showed that incorporating bats can increase the effectiveness of integrated pest management in China.

### 4.1. Dietary Composition and Pest Diversity

In this study, we described the dietary characteristics of four sympatric insectivorous bats. We found that the incidence of agricultural pest consumption by bats was high (exceeding 40%). These included widely distributed species, such as *Cnaphalocrocis medinalis*, *Mythimna separata*, *Sitotroga cerealella*, *Spodoptera frugiperda*, and *H. armigera* (Table 1; Appendix A). These pests feed on various crops, including cotton, corn, peanuts, tobacco, and soybean, which have high economic value [72]. *C. medinalis* causes 70.27 × 10^8^ kg annual rice loss [73], and *S. frugiperda* generally causes yield loss between 15% and 73% in China [74]. The four bat species consumed adult agricultural pests, which might prevent direct crop damage caused by the next generation of pests and indirect crop damage caused by the transmission of fungal diseases. The composition and diversity of prey were greater in our study for each bat species (200 families in 23 orders of arthropods) compared to the prey diversity of bats in areas of intensive agriculture in the Neotropical and Palearctic regions [26,75]. This probably occurred because our study area included farmland and forests, which increased the diversity of insects and provided more diverse food resources for insectivorous bats.

We also discovered the pests *Culex tarsalis* and *Anopheles culicifacies*, which can transmit human and animal diseases, in the diets of the four bat species. Several studies have shown that the population dynamics of Culex mosquitoes may influence the disease dynamics of the West Nile virus (WNV) [76]. In this study, Diptera accounted for a noticeable proportion of the diet of all four bat species (exceeding 10%, except *M. fuliginosus*). The results indicated that the ecosystem service provided by bats via mosquito consumption may be extremely important for protecting public health security in the current global epidemic. Additionally, pests that cause significant damage to forests (such as *Pseudotelphusa paripunctella* and *Dendrolimus kikuchii*) were also found in the diets of the four species of bats, which supports the view that bats provide an important arthropod suppression service to the productivity of forestry [77]. Our study showed that bats consume many types of pests, and measuring the effectiveness of bats in biological control is important, besides documenting the abundance and species of insect pests. Researchers should consider using methodologies that can be used to estimate the impact of bat predation on pest population dynamics. There are a number of studies describing the role of bats for pest control [78,79], but without estimating the real abundance of pest insects, further research should conduct field experiments (exclusion experiments) to measure the real pest abundance. Such information can provide a more comprehensive understanding of the role of bats in pest control. Additionally, research on specific crops for accurately quantifying the value of bats in pest control for agricultural systems is lacking in China [80].

### 4.2. The Relationships between Insect Resources and Bat Activity

Several studies have shown that bat activity is affected by the variation in insect resources, especially Lepidopteran insects [81,82]. Our results showed that the sample sites where bats were most active (i.e., sites with the highest number of passes and the longest duration of activity per night) were those with the highest abundance and diversity of insects. As the diversity and abundance of insects and the abundance of Lepidopteran insects increased, the duration of bat activity increased accordingly, which indicated that the bats responded to changes in insect resources. The number of passes by bats was also higher at sample sites with greater insect abundance. Additionally, although the overall difference in bat activity among the six sample sites was small, the bat activity of sample site 1 was significantly higher than that of sample site 3. The reason for this difference in bat activity may be that sample site 3 is located in a mixed landscape of farmland and residential areas, while sample site 1 is mixed with three landscapes of farmland, residential areas and woodland. The landscape structure of sample site 1 is more complex, and the number and diversity of insects are higher, resulting in an increase in bat activity intensity. Our findings were similar to those of previous studies, where it was reported that the emergence of residential areas might negatively affect bat activity [83]. Insectivorous bats are generally more active and forage in farmland [84,85], and the emergence of forest landscapes might correspondingly increase the activity of bats [86]. Bats are generally opportunistic foragers and feed on diverse prey taxa. Prey availability strongly influences their activity [11], and the pests that are active in agroecosystems can significantly contribute to bat diets. In this study, we did not consider the temporal relevance of bats to insect resources; however, other studies have confirmed their temporal response [12]. Thus, the functional response of bat populations to insect pests may help to monitor and manage sudden pest outbreaks in agricultural systems.

### 4.3. Effects of Species Traits on Dietary Niche among Bat Species

The phenotypic differences among species affect their diet and changes in their niche width [87,88,89]. We found that the width of the dietary niche narrowed as the body size of the four bat species increased, which matched the results of dietary analyses, where larger bats were found to have a lower prey diversity. This occurred probably because larger bats usually prefer to consume larger insects [90]. Larger bats have less maneuverability than smaller bats [91], which makes it difficult for them to feed on small insects. Although the four species were found in the same area, there was a low overlap of dietary ecological niches between *T. melanopogon* and the other three species of bats. Specifically, the dietary niche differentiation of *T. melanopogon* and *H. armiger* was significant. Additionally, species traits may be important factors influencing niche differentiation of insectivorous bats, as these determine their foraging habitat, spatial use, and prey consumption patterns [91,92,93]. Previous studies investigated the relationships only among bat morphology, acoustic characteristics, and foraging habitat, and ignored the direct relationships between these characteristics and prey types [94,95]. In our study, small insects, such as those belonging to Mesostigmata, were negatively correlated with the peak frequency of bats, which was because bats with low-frequency echolocation calls cannot easily detect small insects. Large bats usually emit lower-frequency echolocation calls, have stronger jaws, and can consume harder prey more efficiently than those with gracile jaw morphology [96]. Therefore, they usually feed on larger prey. For example, the large species *H. armiger* had a considerably larger proportion of Coleoptera insects in their diet. The differences in the bandwidth and duration of the echolocation of bats can also affect their predation [97]. In our study, the bandwidth was significantly positively correlated with the number of bats consuming insects belonging to Lepidoptera and Mesostigmata. The duration of echolocation calls in bats was significantly negatively correlated with the number of Orthoptera and Hymenoptera and significantly positively correlated with Mantodea. Thus, the differences in morphology and echolocation calls among the four bat species contributed to the variation in their dietary patterns. We found variations not only between separate species but also within individuals of the same species. Although the diets of these four species of bats overlapped to some extent, species-specific prey preferences indicated that the bat species could co-exist, which improved the ability of bat communities to consume insect prey and even pests.

### 4.4. Implications for Pest Control Services and Bat Conservation

As bats are generalist predators, they can respond to pest outbreaks, track and utilize pest resources, and play an important role in pest suppression in agricultural systems [11,98]. Besides their effect via direct predation, bats also have a top-down effect on crops, reducing crop damage and decreasing the cost of pesticide application [82,99]. However, due to the COVID-19 pandemic, bats in many countries suffered unprecedented persecution, and bat populations have declined considerably in the 21st century [100,101,102]. Therefore, bat-friendly agricultural landscape management strategies need to be promoted. We make the following specific recommendations:

(1) Roost conservation

In our study, the bats that preyed on the widest range of agricultural pests were *A. stoliczkanus* and *M. fuliginosus*, both of which are cave-dwelling species; protecting natural bat roosts and improving the management of caves can be effective in conserving bat populations. In areas where roosts have been destroyed, they can be replaced with artificial roosts, such as bat boxes. Artificial roosts can attract some bats to perform their pest control role [13,103]. However, the use of artificial roosts in Asian countries is still in its infancy and lacks practice and testing in agricultural systems [104].

(2) Landscape conservation

In the agricultural ecosystem, with the increase in landscape heterogeneity, the activity of bats will increase accordingly [105,106]. Protecting the lakes, shrubs, and other landscapes around the farmland can increase biological pest control [107]. Additionally, the protection of patches of forests in the matrix surrounding farmland is important for bats that forage in woodlands, such as *H. armiger*, and also for facilitating bat predation on forestry pests.

(3) Legislation

Legislating to protect bat roosts is an essential way of helping to maintain populations. Several countries, such as Brazil, have already applied laws to protect some ecosystems, including caves [108,109]. In China, the People’s Republic of China Forest is now in force, and the greening of the country’s ground cover with vegetation is gradually increasing [110]. Vegetation protection is very important for arboreal bats and can provide them with enough habitat to choose [111]. However, most of the caves in China do not belong to the current national nature reserve system. Cave ecosystems receive little support from the government. Caves are the habitat of many bat populations. In order to effectively protect bats, it is necessary to pass the legislative protection of cave resources as soon as possible.

(4) Pesticide management

Pesticides are widely used in conventional and integrated pest management farming systems, which may negatively affect bats [112]. Adjustments to pesticide spraying, such as the type of pesticide applied and the frequency of pesticide application, are also necessary to use bats more effectively as pest control agents.

## 5. Conclusions

In this study, we showed that the four insectivorous bats regularly preyed on agricultural pests, and the activity of bats was affected by changes in insect resources. The dietary niche differentiation of the four bat species led to an increase in the type and abundance of insect prey and pests consumed. We found that the diet of bats was correlated with their morphological and echolocation traits. Our findings showed the important ecological role that bats play in agricultural systems. Our results highlighted the need to incorporate bat conservation into integrated pest management programs across different agricultural systems and provide recommendations for it. Future studies need to include more individual characteristics (such as sex and physiological status) to determine the effect of bats on prey selection and their contribution to the differentiation of dietary niches. Additionally, the interaction between bats and pests of specific crops needs to be considered, and more field experiments are needed to quantify the proportion of pests preyed upon by bats as a proportion of all agricultural pests to further clarify the pest control role of bats.

## Figures and Tables

**Table 1:** The top 15 prey species were detected from the fecal samples of four bat species. (Agricultural pests: **, Other pests: *, Non-pests: /).

Scientific Name	Order	Family	FOO	Pest	Damage Stage
*Hylesia pauper*	Lepidoptera	Saturniidae	0.482	**	Fruit-bearing, ornamental and forest plants
*Bembina albinotata*	Coleoptera	Scarabaeidae	0.434	*	Trees (*Terminalia*…)
*Mythimna separata*	Lepidoptera	Noctuidae	0.385	**	Agricultural plants (maize…)
*Spodoptera frugiperda*	Lepidoptera	Noctuidae	0.337	**	Grasses and grain crops
*Spodoptera mauritia*	Lepidoptera	Noctuidae	0.337	**	Various grasses (rice, wheat…)
*Dendrolimus punctatu*	Lepidoptera	Lasiocampidae	0.337	*	*Larix, Picea* and *Pinus* species
*Hypomecis lioptilaria*	Lepidoptera	Geometridae	0.337	*	Trees (oak, birch…)
*Inopsis funerea*	Lepidoptera	Erebidae	0.337	/	/
*Eilema plana*	Lepidoptera	Erebidae	0.337	/	/
*Bradina diagonalis*	Lepidoptera	Crambidae	0.337	**	Various plants (cabbage, eggplant…)
*Leucophenga brevivena*	Diptera	Drosophilidae	0.337	**	Mushrooms and fruit
*Holotrichia serrata*	Coleoptera	Scarabaeidae	0.337	**	Sugarcane, vegetables, groundnut and coconut
*Spodoptera exigua*	Lepidoptera	Noctuidae	0.289	**	Cereals (rice, wheat…)
*Scobura cephaloides*	Lepidoptera	Hesperiidae	0.289	**	Gramineae species (*Indocalamus*…)
*Phaeochrous emarginatus*	Coleoptera	Hybosoridae	0.289	/	/

**Table 2:** A summary of the linear model used to assess the influence of the abundance of insects (AI) and richness of Diptera (DipR) on the number of bat passes. * *p* < 0.05.

	Estimate	SE	*t*	*p*
(Intercept)	−15,219.45	3984.94	−3.819	0.0316 *
AI	2867.75	632.89	4.531	0.0201 *
DipR	−41.83	54.56	−0.767	0.4991

**Table 3:** A summary of the linear model used to assess the influence of the Shannon diversity of insects (HI), the abundance of Lepidoptera (LepA), and the abundance of insects (AI) on the duration of bat activity. ** *p* < 0.01.

	Estimate	SE	*t*	*p*
(Intercept)	−74,437.467	2968.773	−25.07	0.0016 **
HI	4576.271	292.078	15.67	0.0041 **
LepA	13.113	1.063	12.34	0.0065 **
AI	9282.988	424.587	21.86	0.0021 **

**Table 4:** Pianka’s niche-overlap index of the four species of bats.

	*H. armiger*	*T. melanopogon*	*A. stoliczkanus*	*M. fuliginosus*
*H. armiger*	0	0.158	0.627	0.667
*T. melanopogon*	0.158	0	0.302	0.351
*A. stoliczkanus*	0.627	0.302	0	0.677
*M. fuliginosus*	0.667	0.351	0.677	0

## Data Availability

The data presented in this study are available from the corresponding author upon reasonable request.

## Supplementary Materials

The following supporting information can be downloaded at: https://www.mdpi.com/article/10.3390/ani14050815/s1.

## Appendix A

The prey species were detected from fecal samples of four bat species (Supplementary Materials).

## Appendix B

**Table A1:** The results of the pairwise permutational multivariate analysis of variance (PERMANOVA) for dietary niche composition of four species of bats; *p*-values determined by Bonferroni correction.

Comparison	Sum of Squares	Pseudo-F	*R* ^2^	*p*	*Padj*
*H. armiger* vs. *T. melanopogon*	1.408	3.421	0.099	0.001	0.006
*H. armiger* vs. *A*. *stoliczkanus*	0.15	2.050	0.06	0.014	0.084
*H. armiger* vs. *M. fuliginosus*	0.729	1.816	0.055	0.023	0.138
*T. melanopogon* vs. *A. stoliczkanus*	0.721	1.612	0.047	0.022	0.132
*T. melanopogon* vs. *M. fuliginosus*	0.689	1.558	0.046	0.049	0.294
*A. stoliczkanus* vs. *M. fuliginosus*	0.465	1.062	0.031	0.327	1
